# Supplementary material for: Acid-Triggered Release of Eugenol and Fluoride by Desensitizing Macro- and Nanoparticles
Source: J Funct Biomater. 2023 Jan 11;14(1):42. doi: 10.3390/jfb14010042 (PMC9863838; doi:10.3390/jfb14010042)
Supplement: Supplementary file 1 [file jfb-14-00042-s001.zip › jfb-2119428-supplementary.pdf]

Equipment and materials used

Calcium acetate, calcium carbonate, calcium chloride, Casein, disodium hydrogen phosphate, eugenol, absolute ethanol, and polyethylene glycol methyl ether MW=550 were purchased from Sigma-Aldrich. Polyethylene glycol (PEG-300), potassium chloride, sodium bicarbonate, sodium dihydrogen phosphate, phosphoric acid, sodium fluoride, sodium chloride, sodium hydroxide, and Pierce™ BCA Protein Assay Kit were purchased from Thermo Fisher Scientific. Biotene® dry mouth oral rinse (artificial saliva) was purchased from GSK. All the reagents were analytical grade.

Scanning Electron Microscope. Zeiss Sigma Field Emission Scanning Electron Microscope (SEM), accelerating voltage of 4 kV (Carl Zeiss Microscopy, LLC, White Plains, NY, USA)

Powder X-ray diffraction. Rigaku Ultima IV XRD Diffractometer (Rigaku Analytical Devices, Wilmington, MA, USA), Cu Ka radiation (λ = 1.540A, 40 kV, 44 mA)

FTIR spectrometer. IFS Equinox 55 Spectrometer, Bruker.*

UV-Vis Spectrophotometer. Varian Cary 50 UV-Vis Spectrophotometer, Agilent.

Fluoride meter. ExStick FL700, Extech

Time profiles of release of eugenol and fluoride from materials

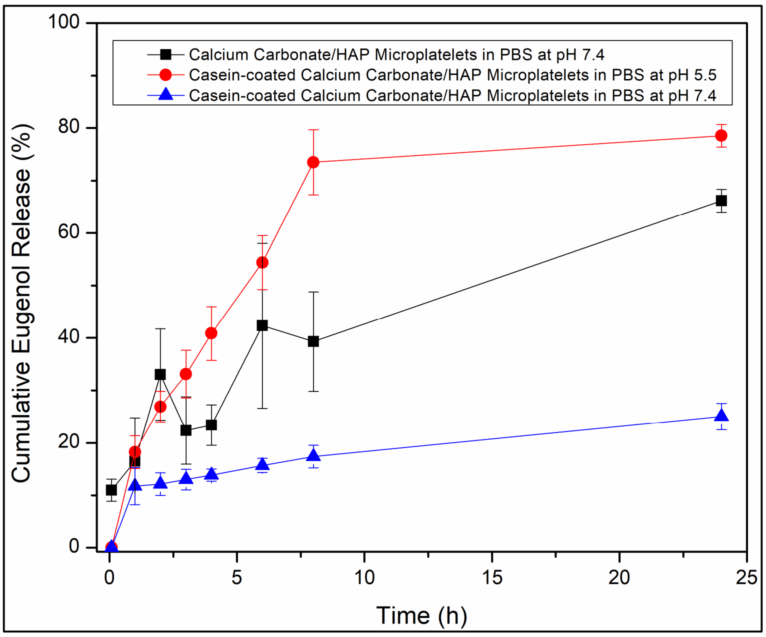

Figure S1. Cumulative eugenol release profiles from calcium carbonate/hydroxyapatite microplatelets in phosphate buffer saline at pH 7.4 (black line). Cumulative eugenol release profile from casein-coated calcium carbonate/hydroxyapatite microplatelets in phosphate buffer saline at pH 7.4 (blue line). Cumulative eugenol release profile from casein-coated calcium carbonate/hydroxyapatite microplatelets in phosphate buffer saline at pH 5.5 (red line).

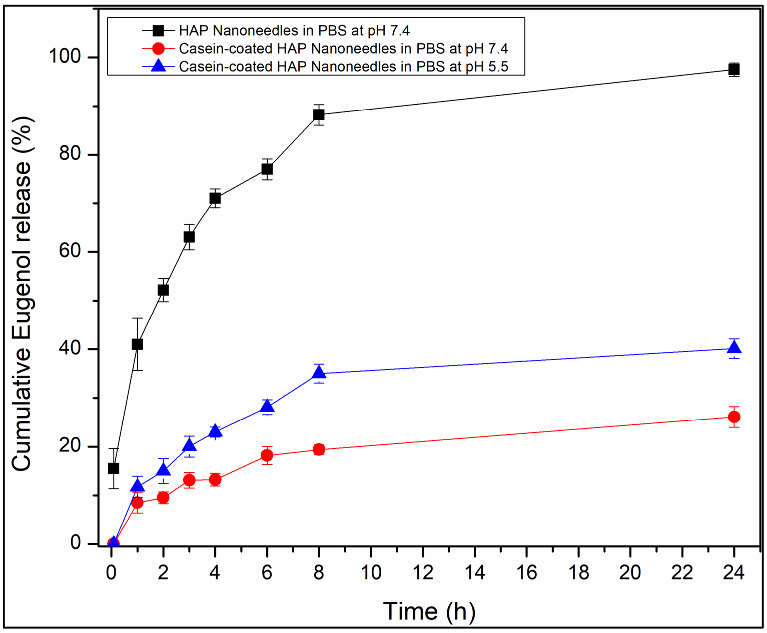

Figure S2. Cumulative eugenol release profiles from hydroxyapatite nanoneedles in phosphate buffer saline at pH 7.4 (black line). Cumulative eugenol release profile from casein-coated hydroxyapatite nanoneedles in phosphate buffer saline at pH 7.4 (red line) and pH 5.5 (blue line).

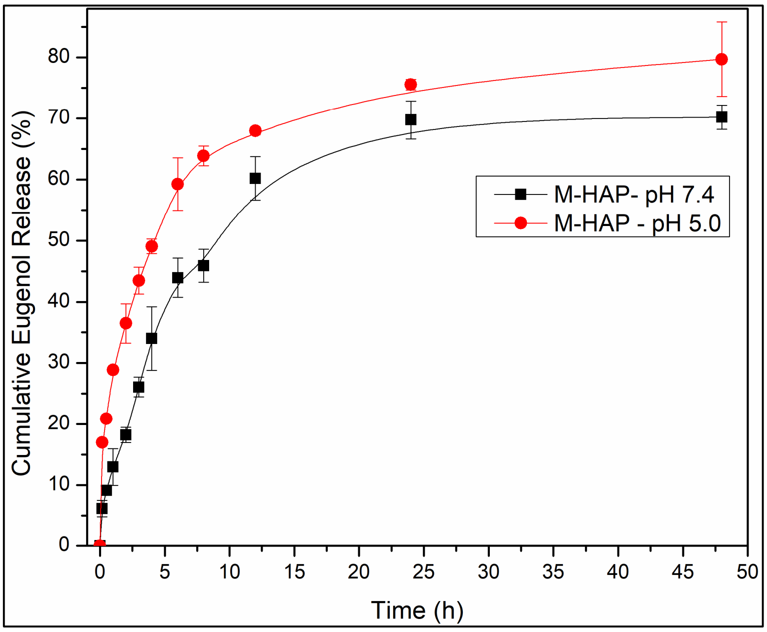

Figure S3. Cumulative eugenol release profiles from mesoporous hydroxyapatite particles in phosphate buffer saline at pH 7.4 (black line) and at pH 5.5 (red line).

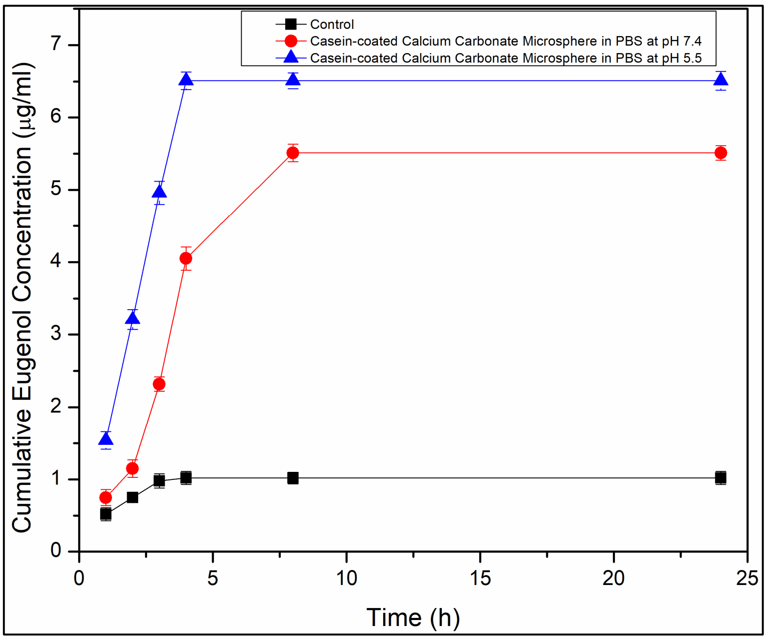

Figure S4. Cumulative eugenol release profiles from casein-coated calcium carbonate microspheres in phosphate buffer saline at pH 7.4 on dentin (red line). Cumulative eugenol release profile from casein-coated calcium carbonate microspheres in phosphate buffer saline at pH 5.5 on dentin (blue line). Cumulative eugenol release profile from eugenol-treated dentin (black line).

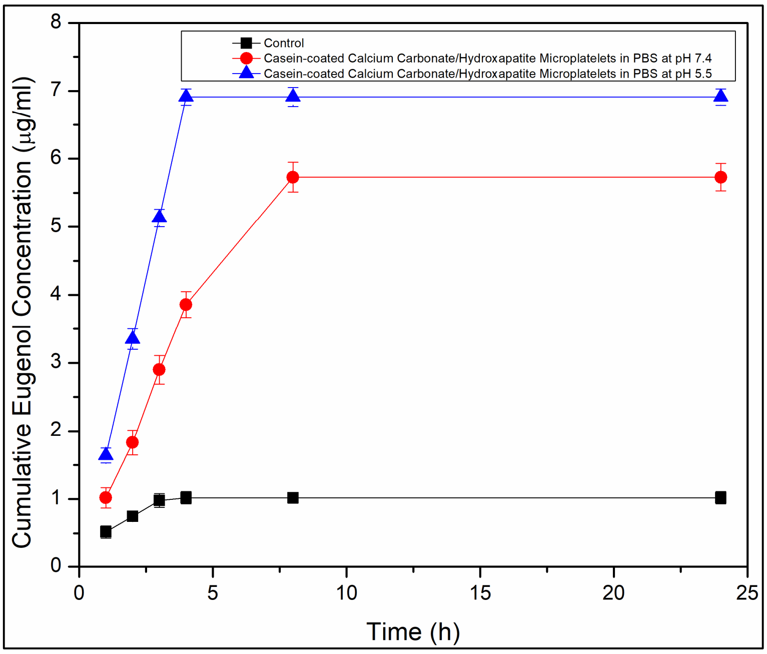

Figure S5. Cumulative eugenol release profiles from casein-coated calcium carbonate/hydroxyapatite microplatelets in phosphate buffer saline at pH 7.4 on dentin (red line). Cumulative eugenol release profile from casein-coated calcium carbonate microspheres in phosphate buffer saline at pH 5.5 on dentin (blue line). Cumulative eugenol release profile from eugenol-treated dentin (black line).

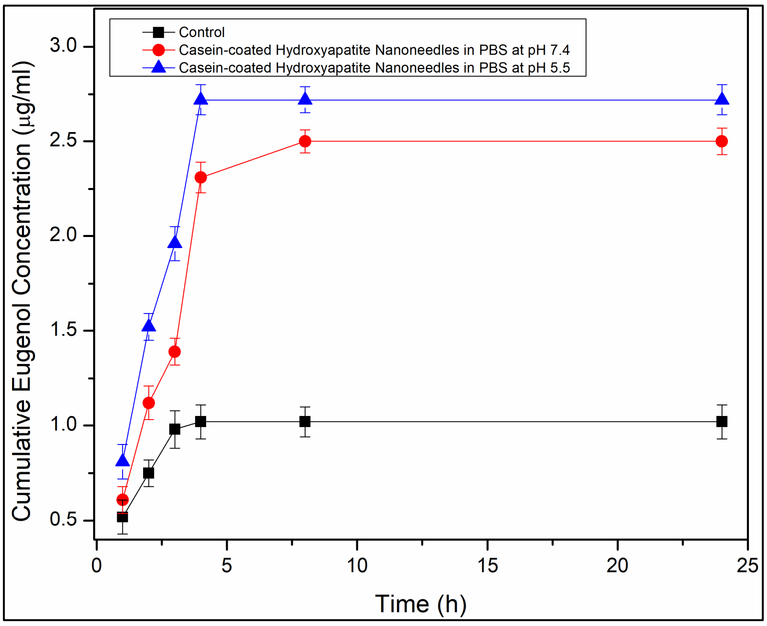

Figure S6. Cumulative eugenol release profiles from casein-coated hydroxyapatite nanoneedles in phosphate buffer saline at pH 7.4 on dentin (red line). Cumulative eugenol release profile from casein-coated calcium carbonate microspheres in phosphate buffer saline at pH 5.5 on dentin (blue line). Cumulative eugenol release profile from eugenol-treated dentin (black line).

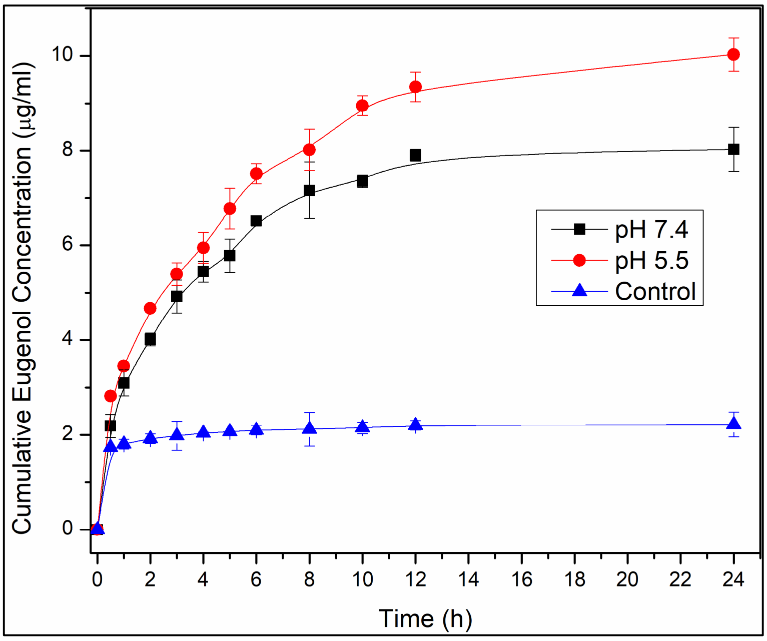

Figure S7. Cumulative eugenol release profiles from mesoporous hydroxyapatite particles in phosphate buffer saline at pH 7.4 on dentin (black line). Cumulative eugenol release profile from casein-coated calcium carbonate microspheres in phosphate buffer saline at pH 5.5 on dentin (red line). Cumulative eugenol release profile from eugenol-treated dentin (blue line).

Protocol for BCA protein analysis

1. The working reagent was prepared by mixing 50 mL of Reagent A with 1mL of Reagent B from the BCA analysis kit.

2. The working reagent (2.0 mL) was well mixed with 0.1 mL of the casein analyte, incubated at 37 ^{o}C for 30 min, and cooled to room temperature.

3. The *absorbance* of the mixture was measured at 562 nm and corrected for the absorbance of a blank sample.

4. A five-point calibration line was plotted for casein at the concentration range of 20.0–125.0 μg/ml to determine the *Intercept* and *Slope*. The amount of conjugated casein in the analyte was calculated by the following equations:

Equation 1: Concentration of non-conjugated casein μg/mL= (Absorbance + Intercept)/Slope

Equation 2: Mass of casein non-conjugated to particles(μg) = Concentration x Dilution factor

Equation 3: Mass of casein conjugated to particles(μg) =Total given mass of casein (μg) - Mass of casein non-conjugated to particles(μg)

Equation 4: Conjugated casein content (%) = (Mass of casein conjugated to particles x 100)/ Mass of the particles.

Table S1. The amount of casein conjugated with calcium carbonate/hydroxyapatite-based particles.

Particle Type	Casein conjugation capacity
Calcium carbonate microsphere	7.2 μg±1.1 μg eugenol/1 mg particles
Calcium carbonate nanosphere	5.6 μg±1.3 μg eugenol/1 mg particles
Hydroxyapatite nanoneedles	5.8 μg±1.1 μg eugenol/1 mg particles
Calcium Carbonate/Hydroxyapatite Microplatelets	5.0 μg±1.2 μg eugenol/1 mg particles

Quantification of eugenol loading

The calibration line was plotted for solutions of eugenol in PBS buffer 7.4: 1, 0.25, 0.2, 0.15, 0.10, 0.075, 0.05, 0.025, 0.010, 0.005, 0.001 mg/mL. All experiments were replicated three times. The amount of unbound eugenol was calculated by **Equations 1-2** based on the observed absorbance and the parameters of the calibration line: Slope and Intercept.

Equation 1: Concentration of unbound eugenol mg/mL = (Absorbance + intercept) /Slope

Equation 2: Percentage of the bound eugenol (%) = Total amount of eugenol loaded to the particles – the amount of unbounded eugenol) x 100 / Total amount of eugenol loaded to the particles

Table S2. Capacity of particles to eugenol (mg of eugenol per 1 mg of particles).

Particle Type	Eugenol loading capacity
Casein-coated calcium carbonate microsphere	17 μg±1 μg
Hydroxyapatite nanoneedle	22 μg±1 μg
Casein-coated hydroxyapatite nanoneedle	20 μg±1 μg
Calcium carbonate/hydroxyapatite microplatelet	17 μg±1 μg
Casein-coated calcium carbonate/hydroxyapatite microplatelet	15 μg±1 μg
Mesoporous hydroxyapatite	22 μg±1 μg

Quantification of eugenol release

All experiments were replicated three times. The amount of released eugenol was calculated by **Equations 3-5** based on the observed absorbance and the parameters of the calibration line: Slope and Intercept.

Equation 3: Concentration of released eugenol mg/mL = (Absorbance + Intercept) /Slope

Equation 4: Amount of eugenol (mg) = Concentration x Dissolution Volume x Dilution factor

Equation 5: Cumulative percentage of the released eugenol (%) = (Concentration of eugenol released at time t + Σ withdrawn eugenol before time t) x 100) / Total amount of eugenol loaded to the particles

Quantification of fluoride loading

Equation 1: Amount unbounded of fluoride (ppm) = Concentration (ppm) x Dilution factor

Equation 2: Amount unbounded of fluoride (ppm) =Total given amount of fluoride (ppm) - Amount unbounded of fluoride (ppm)

Quantification of fluoride release

Equation 1: Amount of fluoride (ppm) = Concentration (ppm) x Dilution factor

Equation 2: Cumulative released of fluoride (ppm) = Concentration of fluoride released at time t + Σ withdrawn fluoride before time t

Preparation of dentin Tooth samples were collected, prepared, and cleaned by the previously reported method [18]. The enamel layer of the tooth specimens was removed to aid in sample processing, except for one sample where the enamel was intentionally preserved for comparison. A tooth was mounted on a Beuhler Isomet Slow-speed saw, sectioned into 2.5 mm × 2.5 mm × 0.5 mm mid-coronal dentin slices with a diamond blade, rinsed with nanopure water, and ultrasonicated for 1 min in 0.2 N citric acid to remove saw smears and debris. The presence of open dentinal tubules in the dentin samples was confirmed by SEM imaging.

Application of particles to dentin A slice of dentin was gently brushed (1 min from each side) with a paste of 100 mg of particles and 1 mL of water. Each side of the dentin was rinsed with water for 10 s, and the slice was sonicated in water for 30 s. The samples were rinsed with water for 10 s for each side and kept on the air.

Loading particles with eugenol in a suspension A 5 mg portion of particles and 1.0 mL of 0.2 mg/mL eugenol in PBS buffer pH 7.4. was mixed on a vortex for 5 min, placed in a Roto-Mini rotating mixer with 24 RPM for 24 h, and centrifuged at 8000 rpm for 6 min. The supernatant A was collected. The precipitate was mixed with 0.5 mL of

PBS buffer pH 7.4, vortexed for 2 min, and centrifuged at 8000 rpm for 6 min. The supernatant B was mixed with supernatant A, and the concentration of unbound eugenol was determined by absorbance at 280 nm.

Release of eugenol from suspension a. A 5 mg portion of particles loaded with eugenol and 1.0 mL of PBS buffer pH 7.4 or PBS pH 5.5 was mixed on a vortex for 5 min in a microcentrifuge tube. b. The tube was placed in a mixer for Time and centrifuged at 8,000 rpm for 6 min. A 200 μL portion of the supernatant was collected, and 200 μL of PBS buffer was added to the tube. The concentration of the released eugenol was determined by absorbance at 280 nm.

c. Step b. was performed for 30 min and was repeated for the following cumulative values of the variable Time: 30 min, 1 h, 2h, 3h, 4h, 6h, 8h, 24 h.

Loading particles with fluoride in a suspension of casein-coated calcium carbonate microspheres A 10 mg portion of particles and 1.0 mL of 1 mg/mL or 1000 ppm fluoride solution in nanopure water was mixed on a vortex for 5 min, placed in a Roto-Mini rotating mixer with 24 RPM for 2 h and centrifuged at 8,000 rpm for 6 min. The supernatant A was collected. The precipitate was mixed with 1 mL of nanopure water, vortexed for 2 min, and centrifuged at 8000 rpm for 6 min. Then, supernatant B was collected. After that, supernatant A and B were mixed and diluted to 20 ml with nanopure water. Again, 1 mL of the diluted solution was taken and diluted to 20 mL with nanopure water. Then the solution was read by a fluoride meter, and amount of the unbounded fluoride was calculated according to equations 1-2. Then fluoride-loaded particles were conjugated with casein according to procedure 4.4.

Release of fluoride from the suspension of casein-coated calcium carbonate microspheres a. A 10 mg portion of particles loaded with fluoride and 12 mL of PBS buffer pH 7.4 or PBS Buffer pH 5.5 or artificial saliva was mixed on a vortex for 5 min in a microcentrifuge tube. b. The tube was placed in a tube holder. A 12 ml portion of the supernatant was collected, and 12 mL of dissolution medium was added to the tube. Next, 1 ml supernatant solution was taken and diluted to 10 ml with nanopure water. Again 1 ml diluted solution was taken and diluted to 20 ml with nanopure water. Then, the concentration of the released fluoride was determined by a fluoride meter. c. The step b. was performed for 1h and was repeated for the following cumulative values of the variable Time: 1 h, 2h, 3h, 4h, 6h, 8h, 24h. All experiments were replicated three times. The amount of released fluoride was calculated by Equations 1-2 based on the observed concentration (ppm) reading with the fluoride meter.

Loading eugenol on dentin slices A dentin slice was placed in 15 mL of the eugenol solutions (0.1g/mL in PBS buffer, pH 7.4). The solution was gently magnetically stirred (250 rpm) for 24 h at room temperature. The drug-loaded specimen was collected with a spatula and washed with PBS buffer (pH 7.4) two times to remove the surface adsorbed eugenol.

Release of eugenol from dentin slices a. A dentin slice loaded with applied eugenol-carrying particles (step 4.6) or eugenol (step 4.9) was placed in 1.0 mL of PBS buffer pH 7.4 or pH 5.5 for ***Time***. A 200 μL portion of the supernatant was collected, and 200 μL of PBS buffer was added to the tube. The concentration of the released eugenol was determined by absorbance at 280 nm. c. The step a. was performed for

30 min and was repeated for the following cumulative values of the variable Time: 30 min, 1 h, 2h, 3h, 4h, 6h, 8h, 12 h, 24 h. The experiment was repeated 3 times for different dentin slices. The amount of released eugenol was calculated by Equations 3-4 based on the observed absorbance and the parameters of the calibration line: Slope and Intercept. The concentration of eugenol was calculated by Equation 5.
